# Financial toxicity, social support, and negative emotions among caregivers of children with cancer: a cross-sectional study in Western China

**DOI:** 10.3389/fpubh.2025.1677962

**Published:** 2026-01-07

**Authors:** Xuan Chen, Sufang Tan, Yuan Li, Qiurong Chen, Hongju Dai

**Affiliations:** 1 Department of Nursing, West China Second University Hospital, Sichuan University, Chengdu, China; 2 Department of Hematology, West China Second University Hospital, Sichuan University, Chengdu, China; 3 Key Laboratory of Birth Defects and Related Diseases of Women and Children (Sichuan University), Ministry of Education, Chengdu, China; 4 Department of Neonatology, West China Second University Hospital, Sichuan University, Chengdu, China

**Keywords:** child, neoplasms, caregivers, financial stress, social support, anxiety, depression

## Abstract

**Purpose:**

To assess the level of financial toxicity (FT) experienced by carers of children with cancer in Western China, identify associated factors.

**Methods:**

A cross-sectional survey was conducted at a large tertiary hospital (*N* = 304 carers). Data included sociodemographic, Comprehensive Score for Financial Toxicity – Patient-Reported Outcome Measure (COST-PROM), perceived social support (PSSS), and anxiety/depression (HADS). Univariate analyses were employed to compare FT scores across groups, Pearson correlations to examine associations between continuous variables, and hierarchical multiple linear regression analysis to identify predictors of FT.

**Results:**

The mean COST-PROM score was 12.24 (SD = 8.44), indicating moderate FT. Elevated FT demonstrated significant correlations with heightened anxiety (*r* = −0.44), depression (*r* = −0.47), and diminished total social support (*r* = 0.32) (all *p* < 0.01). The final regression model (Adjusted R^2^ = 0.309, *p* < 0.001) revealed that factors significantly associated with increased FT included: carers’ employment change (*β* = −0.113), out-of-pocket medical expenses exceeding 40% of annual household income (*β* = −0.112), and the presence of loans (*β* = −0.135). Conversely, the perception that family income was sufficient to cover treatment costs (β = 0.257) and greater perceived support from friends (PSSS; *β* = 0.234) were significantly associated with decreased FT. In addition, both anxiety and depression scores were significantly correlated with higher FT.

**Conclusion:**

Carers in this sample experience moderate FT, which is strongly linked to economic hardship, and diminished social support (particularly from friends). Interventions should address modifiable factors like social support, anxiety, depression, alongside financial navigation and psychological support services.

## 1 Introduction

Childhood cancer represents a significant global health challenge, with a concerning trend of rising incidence and mortality projected, particularly in low- and middle-income countries (LMICs) (1, 2). Globally, approximately 400,000 children and adolescents (0 ~ 19 years) are diagnosed with cancer annually, and survival rates unfortunately lag in LMICs, where over 80% of these cases occur (2) in China, cancer is the second leading cause of pediatric death, with an estimated incidence of 147 cases per million children annually (3). Although advancements in medical technology have improved 5-year survival rates for certain conditions, such as acute lymphoblastic leukemia (exceeding 90% in developed countries) (4), the intensive and prolonged nature of treatment poses immense challenges for affected families (5, 6). For example, a recent national survey in China indicates that the average treatment cost for a child with leukemia ranges from $15,000 to $30,000, creating a significant financial burden (5). In Western China, including Sichuan Province where this study was conducted, medical resources are concentrated in a few major cities, forcing families to travel long distances for treatment, thereby increasing their economic burden. Furthermore, the region’s relatively underdeveloped economy and lower average incomes further exacerbate these financial pressures.

Beyond the direct medical aspects, cancer treatment often triggers a cascade of adverse financial consequences for families, a phenomenon increasingly recognized as ‘financial toxicity’ (FT) (7). FT encompasses not only the direct financial burden (e.g., out-of-pocket costs, direct costs of care) but also the indirect financial (e.g., transportation, lodging during treatment, and others) distress experienced by patients and their families (8). In pediatric oncology, the financial strain can be particularly acute. The research found that low-income caregivers of children with cancer reported more FT than high-income caregivers (9). In low- and middle-income countries, cancer patients and caregivers spend 42% of their annual income on out-of-pocket expenses, compared to 16% in high-income countries (10). Carers, typically parents, often face difficult choices between maintaining employment and providing essential care, leading to significant rates of job loss or modification (11–13). These disruptions, coupled with indirect costs for treatment, travel, accommodation, and supportive care, contribute to widespread household material hardship, which can emerge early in treatment and persist long after its completion (14–17). This economic burden is linked to poorer quality of life and potentially compromised access to or adherence to care (7, 18, 19), disproportionately affecting vulnerable families (16).

Carers are central to the child’s cancer journey, navigating complex medical decisions, managing symptoms, and coordinating care, often under immense emotional duress (20, 21). This demanding role places them at heightened risk for psychological distress, including anxiety and depression, with mothers often reporting more severe mental health impacts (22–24). Emerging evidence indicates a correlation between psychological distress and FT; for instance, studies in adult cancer populations and those with other chronic illnesses have established that financial difficulties are a significant predictor of both anxiety and depression among patients and their careers, and the severity of financial strain often directly correlates with increased anxiety symptoms (25–27). This relationship, while observed across various adult populations, warrants further exploration within the unique context of carers of children with cancer.

Conversely, social support, encompassing emotional support (e.g., empathy and encouragement), tangible support (e.g., practical assistance with tasks or finances), and informational support (e.g., guidance and advice), is widely acknowledged as a crucial buffer against stress and a promoter of mental well-being (28, 29). For families facing childhood cancer, support from family, friends, and the wider community can be instrumental in alleviating both psychological distress (30) and financial strain (9, 31). Currently, most research on the financial burden of cancer treatment has been conducted among adults in high-income countries (32). Yet, quantitative investigations into how different sources of social support specifically relate to FT among carers of children with cancer, particularly in LMIC contexts, are scarce.

Therefore, this study aimed to address these gaps by: (1) quantifying the level of FT experienced by carers of children with cancer in Western China; (2) examining the correlations between FT, perceived social support, caregiver anxiety, and caregiver depression; and (3) identifying sociodemographic, economic, and treatment-related factors associated with FT in this population.

## 2 Methods

### 2.1 Study design

An observational cross-sectional study design was employed. Data were collected between January and December 2024 at a single, large, tertiary specialized hospital for women and children in Western China. The study was conducted in accordance with the STROBE (Strengthening the Reporting of Observational Studies in Epidemiology) guidelines (33). Data were collected post-admission through a sociodemographic information questionnaire, treatment-related information, Financial Toxicity (FT), Perceived Social Support Scale (PSSS), Hospital Anxiety and Depression Scale (HADS).

### 2.2 Participants

Our researchers used a convenient sampling method, distributed electronic questionnaires to participants in the Pediatric Department Blood Neoplasm Inpatient Ward, and explained the questionnaire items to them to assist their questionnaire filling. Eligible participants were primary carers (parents or legal guardians solely or jointly responsible for care and finances for at least 1 month) of children meeting the following criteria: (1) Under the age of 18 years; (2) Diagnosed with cancer confirmed by histopathology; (3) Undergoing active treatment involving chemotherapy, radiotherapy, or surgery; and (4) The caregiver provided voluntary participation. Carers were excluded if they: (1) had another significant illness in the immediate family (parents and children); (2) had psychiatric or cognitive disorders precluding informed consent or survey completion; or (3) were currently participating in other clinical trials or psychological intervention programs.

### 2.3 Sample size

G∗power 3.1 software was used to prior estimate sample size. The effect size *f*^2^ was set as 0.10, the two-tailed a was set as 0.05, the power (1−*β*) was set as 0.8, and 26 predictors were added. The sample size in this study was calculated 253. After considering a wastage rate of 10%, a minimum sample size of 279 was needed.

### 2.4 Measurements

A structured questionnaire collected data on:

- *Sociodemographic information:* Child’s age, gender, nationality, sibling status; Caregiver’s age, relationship to child, marital status, education level, occupational status, employment status change (e.g., job loss, reduced hours); Spouse’s age, education level, occupational status; Annual household income; out-of-pocket medical expenses (If the time from diagnosis is no more than 1 year, it is the total out-of-pocket cost from diagnosis to the time of collecting the questionnaire; if it is more than 1 year, it is the total out-of-pocket cost in the first year after diagnosis) exceeding 40% of annual household income; Presence of debt; Receipt of family financial support; Receipt of social financial support; Medical insurance type.
- *Treatment-related information:* Diagnosis (leukemia/solid tumors), Length of hospital stay, Surgery (yes/no), Number of emergency department visits.
- *Financial toxicity (FT):* The Chinese version (34) of the Comprehensive Score for Financial Toxicity – Patient-Reported Outcome Measure (COST-PROM) (35) was used to assess FT. This 11-item scale measures subjective and objective FT using a 5-point Likert scale (0 = ‘not at all’ to 4 = ‘very much’). Total scores range from 0 to 44, with lower scores indicating higher FT. Scores are graded: Grade 1 (>26, no impact); Grade 2 (14–25, mild); Grade 3 (1–13, moderate); and Grade 4 (0, high) (36). Items were adapted to refer to the child’s illness in this study. The scale’s reliability was high, with a Cronbach’s alpha of 0.843 and a Guttman’s split-half reliability of 0.833 in our sample.
- *Perceived social support:* The Chinese version of the Perceived Social Support Scale (PSSS) (37) was utilized to measure perceived social support. This 12-item scale measures perceived support on a 7-point Likert scale (1 = ‘strongly disagree’ to 7 = ‘strongly agree’). Total scores range from 12 to 84, with higher scores indicating greater perceived support. PSSS comprises three 4-item subscales assessing support from family, support from friends, and support from significant others. The scale’s Cronbach’s alpha coefficient was 0.90 in the present study.
- *Anxiety and depression:* The Chinese version of the Hospital Anxiety and Depression Scale (HADS) (38, 39) was employed to evaluate symptoms of anxiety and depression. This 14-item scale uses a 4-point Likert scale (0–3). HADS consists of two 7-item subscales: HADS-Anxiety (HADS-A) and HADS-Depression (HADS-D), assessing symptoms in the past month. Subscale scores range from 0–21, with 0–7 indicating non-cases, 8–10 suggesting doubtful cases, and 11–21 indicating definite anxiety/depression (40). Both subscale scores were used in analyses. In the current study, the scale’s reliability was high, with an overall Cronbach’s alpha of 0.884, with a Cronbach’s coefficient of 0.762 for the depression subscale and a Cronbach’s coefficient of 0.831 for the anxiety subscale.

### 2.5 Ethical considerations

Ethical approval was obtained from the Institutional Review Board of West China Second University Hospital, Sichuan University (Approval no. [23H0917]). Subsequently, the first author then explained the purpose of the study to eligible carers of children with cancer and obtained their informed consent before they completed the questionnaire.

### 2.6 Data collection

Five trained research assistants collaborated to approach eligible carers during their child’s hospitalization. After explaining the study purpose, risks, and benefits, and assuring confidentiality and the right to withdraw, written informed consent was obtained. Consenting carers completed the paper-based questionnaire independently in a relatively quiet area on the ward or at the child’s bedside, depending on their preference and convenience. The estimated time for completion was approximately 15–20 min. Research assistants remained available to answer questions and checked returned questionnaires immediately for completeness to minimize missing data.

### 2.7 Data analysis

Data were entered into Excel and analyzed using SPSS version 26.0. Descriptive statistics (means, standard deviations [SD], frequencies, percentages) were used to summarize participant characteristics and scale scores. Specifically, we employed the Kruskal Wallis H test to analyze polytomous variables and utilized the Mann–Whitney *U* test for dichotomous variables. Independent samples t-tests and one-way ANOVA were used to compare mean COST-PROM scores across different categorical demographic and clinical groups. Pearson correlation coefficients (*r*) were calculated to assess linear associations between COST-PROM scores, PSSS (total and subscales) scores, and HADS (anxiety and depression subscales) scores. A hierarchical multiple linear regression analysis was performed to identify independent factors associated with FT. The selection of predictors (i.e., PSSS) was based on previous literature [e.g., (9, 31)]. The selection of covariates, including demographic (age, gender, education, occupational status, nationality, sibling status, relationship to child, marital status, employment status change, annual household income, presence of debt, medical insurance type), was made because they may influence FT (22–24, 41–44) and mitigate any potential confounders and retrospective bias. Variables showing significant associations (*p* < 0.05) in univariate analyses or deemed clinically important were considered for inclusion. Variables were entered in blocks: Block 1 included key sociodemographic and treatment-related factors; Block 2 added PSSS subscale scores (family, friends, others). Standard checks for multicollinearity (Tolerance, Variance Inflation Factor [VIF]) and residual analysis (Durbin-Watson) were performed. Statistical significance was set at *p* < 0.05 (two-tailed).

## 3 Results

### 3.1 Participant characteristics

Of 320 questionnaires distributed, 304 were returned and deemed valid (response rate: 95%). Key characteristics of the participants and their association with FT scores from univariate analyses are presented in Table 1. The mean age of the children was 7.27 (SD = 3.98) years, and 58.2% were male. Leukemia was the most common diagnosis (60.9%). The majority of carers were mothers (81.9%), married (88.2%), with a mean age of 35.08 (SD = 5.82) years. Notably, 72.0% reported employment changes, 82.2% stated family income could not cover treatment costs, 75.7% had out-of-pocket expenses exceeding 40% of household income, and 45.7% reported having debt. Univariate analyses indicated that numerous sociodemographic and economic factors were significantly associated with higher FT. These included aspects such as caregiver and spouse occupational and educational status, employment changes, income adequacy, out-of-pocket expenses, debt, financial support received, medical insurance type, and annual household income (all *p* < 0.05, see Table 1 for specific statistics).

**Table 1:** Sociodemographic and clinical characteristics of caregivers and children, and univariate analysis of financial toxicity (FT) scores (*n* = 304).

Variables	Category	*N* (%)	FT score (means ± SD)	Test stat (t/F)	*p*-value
Child age (years)	0 ~ 6	151 (49.6)	12.73 ± 8.39	0.556	0.574
	7 ~ 12	116 (38.2)	11.63 ± 8.89		
	13 ~ 18	37 (12.2)	12.16 ± 7.19		
Child gender	Female	127 (41.8)	13.09 ± 9.29	1.454	0.147
	Male	177 (58.2)	11.63 ± 7.75		
Nationality	Han	264 (86.8)	12.57 ± 8.54	−1.746	0.082
	Minority	40 (13.2)	10.08 ± 7.55		
Only child	Yes	104 (34.2)	13.47 ± 9.54	−1.725	0.086
	No	200 (65.8)	11.6 ± 7.76		
Medical insurance	None	4 (1.3)	7.25 ± 8.62	6.160	**0.002**
	Urban employees	106 (34.9)	14.44 ± 9.44		
	NCMS (rural)	194 (63.8)	11.14 ± 7.61		
Caregiver relationship	Mother	249 (81.9)	12.26 ± 8.58	0.092	0.927
	Father	55 (18.1)	12.15 ± 7.89		
Marital status	Married	268 (88.2)	12.27 ± 8.45	1.614	0.201
	Divorced/separated/widowed	29 (9.5)	10.79 ± 8.29		
	Others	7 (2.3)	17.14 ± 7.88		
Caregiver occup. status	Full-time	76 (25.0)	15.24 ± 9.33	**0.131**	**<0.001**
	Part-time or unemployed	228 (75.0)	11.24 ± 7.90		
Spouse occup. status	Full-time	130 (42.8)	13.63 ± 8.44	**2.504**	**0.013**
	Part-time or unemployed	174 (57.2)	11.20 ± 8.32		
Caregiver employ. change	Yes	219 (72.0)	10.74 ± 7.99	−5.198	**<0.001**
	No	85 (28.0)	16.12 ± 8.38		
Income covers treatment	Yes	54 (17.8)	20.48 ± 9.08	8.865	**<0.001**
	No	250 (82.2)	10.46 ± 7.16		
OOP > 40% income	Yes	230 (75.7)	11.20 ± 7.59	3.375	**0.001**
	No	74 (24.3)	15.49 ± 10.05		
Caregiver education	Junior high school or below	121 (39.8)	9.93 ± 6.53	6.496	**<0.001**
	Senior high school/technical secondary school	67 (22.0)	11.48 ± 7.24		
	Junior college	54 (17.8)	14.30 ± 8.73		
	Bachelor’s degree or above	62 (20.4)	15.77 ± 10.94		
Spouse education	Junior high school or below	125 (41.1)	9.68 ± 6.61	7.830	**<0.001**
	Senior high school/technical secondary school	76 (25.0)	12.30 ± 7.79		
	Junior college	49 (16.1)	14.65 ± 9.11		
	Bachelor’s degree or above	54 (17.8)	15.89 ± 10.48		
Family financial support	Yes	93 (30.6)	14.82 ± 9.39	−3.345	**0.001**
	No	211 (69.4)	11.10 ± 7.75		
Social financial support	Yes	109 (34.9)	12.99 ± 8.84	2.192	**0.029**
	No	195 (64.1)	10.89 ± 7.54		
Debt	Yes	139 (45.7)	9.41 ± 6.84	5.756	**<0.001**
	No	165 (54.3)	14.62 ± 8.93		
Annual household income (CNY)	≤ 30,000	106 (34.9)	10.10 ± 7.26	17.608	**<0.001**
	30,001 ~ 60,000	113 (37.2)	11.78 ± 7.69		
	60,001 ~ 120,000	54 (17.8)	12.02 ± 7.12		
	≥ 120,001	31 (10.1)	21.61 ± 10.84		
Length of stay (days)	0 ~ 49	140 (46.0)	12.26 ± 8.23	0.947	0.418
	50 ~ 99	98 (32.2)	12.87 ± 8.70		
	100 ~ 149	47 (15.5)	12.06 ± 8.88		
	≥ 150	19 (6.3)	9.32 ± 7.48		
Surgery	Yes	72 (23.7)	12.46 ± 9.23	−0.251	0.802
	No	232 (76.3)	12.17 ± 8.20		
Emergency visits (*N*)	0	150 (49.4)	12.24 ± 8.49	0.147	0.932
	1 ~ 2	84 (27.6)	12.13 ± 8.79		
	3 ~ 5	49 (16.1)	12.80 ± 8.60		
	>5	21 (6.9)	11.38 ± 6.55		

FT Score, COST-PROM Score (Lower score indicates higher financial toxicity); SD, Standard Deviation; NCMS, New Rural Cooperative Medical System; OOP, Out-of-pocket medical expenses. Bold *p*-values indicate statistical significance (*P* < 0.05).

### 3.2 Descriptive statistics and correlations

The mean COST-PROM score indicated moderate FT (Mean = 12.24, SD = 8.44). Mean HADS scores suggested possible anxiety (Mean = 10.97, SD = 4.28) and possible depression (Mean = 10.75, SD = 4.07). The mean total PSSS score was 56.47 (SD = 14.98). Key descriptive statistics and correlations are presented in Table 2. Higher FT showed significant negative correlations with HADS anxiety (*r* = −0.44) and depression (*r* = −0.47) scores, and significant positive correlations with PSSS total (*r* = 0.32) and all PSSS subscale scores (Relatives: *r* = 0.28; Friends: *r* = 0.35; Others: *r* = 0.24), indicating associations between higher FT, poorer mental health, and lower perceived social support (all *p* < 0.01).

**Table 2:** Descriptive statistics and Pearson correlation coefficients for financial toxicity (FT), anxiety, depression, and perceived social support scales (*n* = 304).

Scale/subscale	No. of items	Mean (SD)	1	2	3	4	5	6	7	8
1. FT (COST-PROM)	11	12.24 (8.44)	1							
2. HADS total	14	21.72 (7.85)	−0.49^a^	1						
3. HADS anxiety (HADS-A)	7	10.97 (4.28)	−0.44^a^	0.94^a^	1					
4. HADS depression (HADS-D)	7	10.75 (4.07)	−0.47^a^	0.94^a^	0.76^a^	1				
5. PSSS total	12	56.47 (14.98)	0.32^a^	−0.4^a^	−0.36^a^	−0.4^a^	1			
6. PSSS support from relatives	4	19.92 (5.67)	0.28^a^	−0.37^a^	−0.34^a^	−0.36^a^	0.88^a^	1		
7. PSSS support from friends	4	17.78 (5.62)	0.35^a^	−0.40^a^	−0.34^a^	−0.40^a^	0.90^a^	0.67^a^	1	
8. PSSS support from others	4	18.77 (5.35)	0.24^a^	−0.35^a^	−0.30^a^	−0.35^a^	0.91^a^	0.71^a^	0.77^a^	1

FT Score, COST-PROM Score (Lower score indicates higher financial toxicity); HADS, Hospital Anxiety and Depression Scale; PSSS, Perceived Social Support Scale; SD, Standard Deviation. ^a^*P* < 0.01 (two-tailed).

### 3.3 Factors associated with financial toxicity

Prior to the primary multiple linear regression, a preliminary collinearity diagnostic was conducted. Initial assessments revealed Tolerance values ranging from 0.050 to 0.890 (some values were below the 0.1 threshold) and Variance Inflation Factor (VIF) values from 1.122 to 19.917 (some values exceeded the threshold of 5). Exclusion of the “medical insurance” variable, which presented the highest VIF, yielded improved diagnostic metrics: Tolerance values ranged from 0.298 to 0.898 (all >0.1), and VIFs from 1.114 to 3.351 (all <5). The Durbin-Watson statistic was 1.815. These revised diagnostics indicated the absence of severe multicollinearity among the retained independent variables, thereby satisfying a key assumption for the subsequent regression analysis.

The hierarchical multiple linear regression results examining factors associated with FT are presented in Table 3. The first block, comprising sociodemographic and economic factors, significantly predicted FT and explained 27.0% of its variance (Adjusted R^2^ = 0.270, *F* = 11.163, *p* < 0.001). Finally, the addition of Perceived Social Support Scale (PSSS) subscales in the second block accounted for an additional 3.9% of the variance in FT (∆Adjusted R^2^ = 0.039; Adjusted R^2^ for Block 2 = 0.309, *F* = 10.686, *p* < 0.001). In this final model, factors significantly associated with increased FT (i.e., lower COST-PROM scores) included caregiver employment change (*β* = −0.113, *p* < 0.05), out-of-pocket medical expenses exceeding 40% of annual household income (β = −0.112, *p* < 0.05), and the presence of loans (*β* = −0.135, *p* < 0.05). Conversely, the perception that family income was sufficient to cover treatment costs (*β* = 0.257, *p* < 0.01) and greater perceived support from friends (PSSS; *β* = 0.234, *p* < 0.01) were significantly associated with decreased FT. Therefore, we finally obtained the regression equation between the independent variable and the dependent variable: FT = 10.723 ± 2.199 * out-of-pocket medical expenses exceeding 40% of annual household income + 5.659 * covered treatment costs ± 2.288 * the presence of loans ± 2.124 * caregiver employment change + 1.403 * greater perceived support from friends (PSSS).

**Table 3:** Hierarchical multiple linear regression analysis of factors associated with financial toxicity (COST-PROM score) among caregivers (*n* = 304).

Variable	Step 1			Step 2		
	B	*β*	(95% CI of *β*)	B	*β*	(95% CI of *β*)
Block 1: socioeconomic factors						
Education level of caregiver	−0.122	−0.017	(−1.283, 1.038)	−0.010	−0.001	(−1.143, 1.124)
Education level of the caregiver’s spouses	0.767	0.103	(−0.545, 2.080)	0.459	0.061	(−0.827, 1.745)
Occupational status of the caregiver	0.863	0.044	(−1.298, 3.024)	0.929	0.048	(−1.182, 3.040)
Occupational status of the caregiver’s spouses	−1.308	−0.077	(−3.335, 0.718)	−1.271	−0.075	(−3.248, 0.706)
Income covers treatment (Yes vs. No)	5.917	0.268*^d^*	(3.269, 8.565)	5.659	0.257*^d^*	(3.074, 8.244)
OOP > 40% income (Yes vs. No)	−2.165	−0.110^b^	(−4.169, −0.162)	−2.199	−0.112^b^	(−4.167, −0.230)
Annual household income	0.705	0.081	(−0.515, 1.925)	0.458	0.053	(−0.738, 1.655)
Family financial support (Yes vs. No)	1.564	0.085	(−0.342, 3.470)	0.749	0.041	(−1.142, 2.641)
Social financial support (Yes vs. No)	−0.016	−0.001	(−1.822, 1.789)	0.168	0.010	(−1.601, 1.937)
Loans (Yes vs. No)	−2.557	−0.151*^c^*	(−4.375, −0.740)	−2.288	−0.135^b^	(−4.062, −0.513)
Caregiver employ. change (Yes vs. No)	−2.439	−0.130^b^	(−4.428, −0.450)	−2.124	−0.113^b^	(−4.066, −0.182)
Block 2: PSSS subscales						
Support from relatives				0.559	0.094	(−0.286, 1.405)
Support from friends				1.403	0.234*^c^*	(0.452, 2.355)
Other support				−0.654	−0.103	(−1.682, 0.375)
Model summary						
R^2^	0.296			0.341		
Adjusted R^2^	0.270			0.309		
∆R^2^	0.296			0.045		
F	11.163^d^			10.686^d^		

β = Standardized regression coefficient. Dependent Variable = COST-PROM Score. PSSS, Perceived Social Support Scale; HADS, Hospital Anxiety and Depression Scale; OOP, Out-of-pocket. ^b^*P* < 0.05, ^c^*P* < 0.01, ^d^*P* < 0.001.

## 4 Discussion

This study provides quantitative insights into the financial toxicity (FT) experienced by carers of children with cancer at a tertiary hospital in Western China, highlighting its prevalence and association with sociodemographic factors, social support, and mental health. Carers in this cohort reported moderate levels of FT, with a mean COST-PROM score (12.24) falling within the Grade 3 impact category (36). This level appears more severe than that reported in some studies of adult cancer patients (41), potentially reflecting the prolonged dependency and intensive care needs of children undergoing cancer treatment. Furthermore, our regression analysis identified several key factors significantly associated with FT. Specifically, caregiver employment disruptions, high out-of-pocket medical expenses, and the presence of loans were significantly associated with increased FT. Conversely, the perception that family income was sufficient for treatment costs and greater perceived support from friends emerged as factors associated with lower FT.

Consistent with prior research on objective financial burdens in pediatric oncology (9, 14, 45, 46), this study identified caregiver employment disruptions, substantial out-of-pocket medical expenses (exceeding 40% of annual household income), and the presence of loans as significant predictors of increased FT. Indeed, our hierarchical regression analysis demonstrated that initial socioeconomic and demographic factors accounted for a substantial portion (27.0%) of the variance in FT, laying a foundational understanding of the economic landscape these carers navigate. These findings underscore the considerable economic strain on these families. Specifically, caregiver employment disruptions, such as job loss or reduced work hours to accommodate caregiving responsibilities, directly diminish household income. Compared to high-income countries such as Switzerland (FT = 28.0) (47) and the United States(FT = 22.0) (48), the severity of FT observed in our study is more pronounced. In China, the coverage and protection provided by children’s social medical insurance are relatively limited. Furthermore, the uneven distribution of medical resources leads to cross-regional medical treatment, which further reduces reimbursement rates and increases out-of-pocket expenses for individuals. Substantial out-of-pocket expenditures, can deplete savings and compel families to incur debt, a correlation underscored by the identified association between loans and FT. Collectively, these elements delineate a clear trajectory towards exacerbated financial hardship, highlighting the multifaceted nature of the objective financial burden.

In terms of protective factors, the regression analysis revealed that the perception that family income was sufficient to cover treatment costs (*β* = 0.257, *p* < 0.01) was significantly associated with decreased FT. This intuitively suggests that when a family’s financial resources can adequately meet the demands of treatment, the likelihood or severity of experiencing financial toxicity is reduced. Furthermore, and consistent with existing literature highlighting the buffering role of social networks (9, 41), greater perceived support specifically from friends (PSSS; *β* = 0.234, *p* < 0.05) also emerged as a significant factor associated with lower FT, even after controlling for other variables. Notably, the inclusion of social support variables explained an additional 4.0% of the variance in FT beyond socioeconomic factors, with support from friends being particularly salient. This finding underscores the unique contribution of friend-based support in mitigating financial distress. The standardized beta coefficient for support from friends (*β* = 0.234) indicates that a one standard deviation increase in such support corresponds to a 0.234 standard deviation increase in the COST-PROM score (signifying lower FT). This suggests that support from friends may offer distinct forms of tangible aid, emotional solace, or informational resources that are particularly effective in alleviating the financial burdens faced by these carers, a dimension potentially differing from support received from family members who might be equally enmeshed in the crisis. This specific role of friend support warrants further investigation.

Furthermore, our results underscore the link between caregiver mental health and FT. The Pearson correlation coefficients analysis powerfully demonstrated the impact of mental health, which higher FT showed significant negative correlations with HADS anxiety (*r* = −0.44) and depression (*r* = −0.47)scores. This finding resonates with studies in other populations (26, 27) and suggests a potentially bidirectional relationship: financial strain may exacerbate depressive symptoms, while depression might impair a caregiver’s ability to cope with financial challenges, seek resources, or maintain employment. The high average HADS scores, bordering on clinical significance for both anxiety and depression, emphasize the profound psychological toll on these carers and their connection to their financial well-being. Addressing caregiver mental health is, therefore, not only crucial for their own sake but may also be an important avenue for mitigating FT.

### 4.1 Strengths and limitations

While this study provides valuable regional data on FT among pediatric cancer carers, several limitations must be acknowledged. The cross-sectional design precludes causal inference; future longitudinal research is essential to track FT, support, and mental health over time. Convenience sampling at a single urban hospital limits generalizability; multi-center studies across diverse settings are recommended to enhance external validity. Self-reported data may be subject to recall or social desirability bias; subsequent studies could benefit from incorporating objective financial measures or multiple informants. Furthermore, a deeper understanding could be gained by examining how specific cancer types, treatment protocols, and caregiver coping strategies influence FT and psychological outcomes. Investigating the mechanisms by which friend support mitigates FT, and the potential mediating role of mental health in the link between financial strain and distress, are also important future research directions. Finally, the study’s focus on Western China necessitates broader research in varied socioeconomic contexts to confirm the wider applicability of these findings.

### 4.2 Implications and future directions

Our findings carry significant implications for both clinical practice and future research. Healthcare providers, particularly oncology nurses who maintain frequent contact with affected families, should be acutely aware of the heightened risk of FT among carers of children with cancer. The implementation of routine screening using validated instruments like the COST-PROM can facilitate the early identification of families grappling with substantial financial distress. Our regression analysis pinpoints specific factors associated with increased FT (caregiver employment change, high out-of-pocket medical expenses, and presence of loans) and decreased FT (the perception that family income is sufficient for treatment costs and robust support from friends). These insights should guide the development of targeted interventions. Such interventions ought to be multifaceted, integrating financial navigation assistance, resources for practical support (aimed at reducing out-of-pocket expenditures and promoting employment retention), mental health screening and support for carers, and strategies designed to fortify social support networks, with particular attention to friend support. Nurses are uniquely positioned to assess these vulnerabilities, deliver pertinent education, and direct families to appropriate resources, including social work and psychological services.

Ultimately, addressing FT requires a comprehensive, evidence-based strategy. Future research should prioritize developing and evaluating culturally tailored interventions for carers’ financial and psychosocial needs, including longitudinal and multi-center trials to assess long-term impacts and enhance generalizability. Further exploration of how friend support and mental health interact with financial toxicity is also crucial for refining interventions. Greater attention is also needed to conduct more studies on financial burden in low- and middle-income countries, as well as among children, adolescents, and caregivers, and to implement corresponding healthcare policies and benefits to alleviate their financial strain. Advocating for health policy reforms to reduce the financial burden of cancer care, particularly in resource-limited settings, remains an urgent priority.

## 5 Conclusion

Carers of children undergoing cancer treatment in this Western China sample experience moderate financial toxicity. This financial distress is significantly associated with several economic hardships, including treatment costs covered by income, caregiver employment change, high out-of-pocket medical expenses, the presence of loans, and support from friends. Conversely, having family income perceived as sufficient to cover treatment costs and perceiving greater social support from friends were linked to lower financial toxicity. In addition, both anxiety and depression scores were significantly correlated with higher FT. These findings underscore the critical need for integrated support systems within pediatric oncology settings. Such systems must address not only the child’s medical requirements but also the profound financial and psychosocial challenges confronting their careers. Addressing modifiable factors, such as caregiver depression and anxiety, the strengthening of social support networks (particularly with friends), alongside alleviating direct economic pressures identified in this study (such as employment disruptions and loan burdens), are crucial components in mitigating the pervasive burden of FT.

## Data Availability

The original contributions presented in the study are included in the article/supplementary material, further inquiries can be directed to the corresponding authors.
