# Correction: Financial toxicity, social support, and negative emotions among caregivers of children with cancer: a cross-sectional study in Western China

**DOI:** 10.3389/fpubh.2026.1916088

**Published:** 2026-07-03

**Authors:** Frontiers Production Office

**Affiliations:** Frontiers Media SA, Lausanne, Switzerland

**Keywords:** child, neoplasms, caregivers, financial stress, social support, anxiety, depression

Authors Xuan Chen and Sufang Tan was erroneously omitted as equal contributing authors who share first authorship.

The original version of this article has been updated.

